# Oxidative Stress Induces HSP90 Upregulation on the Surface of Primary Human Endothelial Cells: Role of the Antioxidant 7,8-Dihydroxy-4-methylcoumarin in Preventing HSP90 Exposure to the Immune System

**DOI:** 10.1155/2018/2373167

**Published:** 2018-04-10

**Authors:** Elisabetta Profumo, Brigitta Buttari, Lavinia Tinaburri, Daniela D'Arcangelo, Maurizio Sorice, Antonella Capozzi, Tina Garofalo, Antonio Facchiano, Rita Businaro, Prashant Kumar, Brajendra K. Singh, Virinder S. Parmar, Luciano Saso, Rachele Riganò

**Affiliations:** ^1^Department of Cardiovascular, Dysmetabolic and Aging-Associated Diseases, Istituto Superiore di Sanità, Viale Regina Elena 299, 00161 Rome, Italy; ^2^Laboratory of Molecular and Cellular Biology, Istituto Dermopatico dell'Immacolata-IRCCS, FLMM, Via Monti di Creta 104, 00167 Rome, Italy; ^3^Laboratory of Vascular Pathology, Istituto Dermopatico dell'Immacolata-IRCCS, FLMM, Via Monti di Creta 104, 00167 Rome, Italy; ^4^Department of Experimental Medicine, Sapienza University of Rome, Viale Regina Elena 324, 00161 Rome, Italy; ^5^Department of Medico-Surgical Sciences and Biotechnologies, Sapienza University of Rome, Viale XXIV Maggio 7, 04100 Latina, Italy; ^6^University of Delhi, Bioorganic Laboratory, Department of Chemistry, New Delhi 110 007, India; ^7^Central University of Haryana, School of Chemical Sciences, Mahendragarh, Haryana 123 031, India; ^8^Institute of Advanced Sciences, 86-410 Faunce Corner Mall Road, Dartmouth, MA 02747, USA; ^9^Department of Physiology and Pharmacology “Vittorio Erspamer”, Sapienza University of Rome, Piazzale Aldo Moro 5, 00185 Rome, Italy

## Abstract

We have previously demonstrated that human heat shock protein 90 (HSP90), an intracellular self protein, is the target of cellular and humoral autoimmune responses in patients with carotid atherosclerosis. In this study, we evaluated *in vitro* whether oxidative stress, a feature of atherosclerotic plaque, alters HSP90 expression in endothelial cells, thus inducing surface localization of this molecule and whether the antioxidant compound 7,8-dihydroxy-4-methylcoumarin (7,8-DHMC) is able to prevent oxidative stress-induced alterations of HSP90 localization. By the use of flow cytometry, immunofluorescence, enzyme-linked immunosorbent assay, and semiquantitative reverse-transcription polymerase chain reaction, we demonstrated that exposure of human umbilical vein endothelial cells (HUVEC) to the prooxidant compound H_2_O_2_ upregulated HSP90 surface expression and reduced its secretion without altering HSP90 gene expression and intracytoplasmic protein levels. Pretreatment of HUVEC with 7,8-DHMC prevented H_2_O_2_-induced alterations of HSP90 cellular distribution and secretion. Our results suggest that the strong oxidative conditions of atherosclerotic plaques promote the upregulation of HSP90 surface expression on endothelial cells, thus rendering the protein a possible target of autoimmune reactions. The antioxidant 7,8-DHMC, by preventing oxidative-stress-triggered HSP90 surface upregulation, may be useful to counteract possible autoreactive reactions to HSP90.

## 1. Introduction

Atherosclerosis is a chronic inflammatory disease of the arterial wall characterized by autoimmune responses against self molecules [1]. Atherosclerosis-related antigens include oxidized low-density lipoproteins, apolipoprotein B-100, and heat shock proteins (HSPs) [2]. HSPs are ATP-dependent chaperones involved in multiple activities within the cells [3]. Besides being expressed in cells under physiological conditions, HSPs increase in response to many environmental stressors, including oxidative stress [4], a hallmark of atherosclerosis [5]. Under stress conditions, HSPs are expressed not only within the cells but also on the cell surface and can be released into the extracellular space [6]. HSP90 is one of the most abundant HSPs in mammalian cells, and the abnormal levels of this protein have been observed in malignant cells and inflamed tissues [7]. Therefore, this HSP is particularly considered in the context of treatment of cancer and inflammatory diseases, such as many skin pathologies and autoimmune processes including autoimmune bullous skin diseases, rheumatoid arthritis, and systemic lupus erythematosus [7–9]. In previous studies, we demonstrated that human HSP90, one of the most abundant HSPs in mammalian cells, is the target of immune responses in patients with carotid atherosclerosis and is overexpressed in plaques and sera from the same patients [10, 11]. The observation that H_2_O_2_ treatment induced HSP90 surface expression in the endothelial cell line EAhy-926 led us to hypothesize that the prooxidant microenvironment of atherosclerotic plaque promotes the translocation of HSP90 from the cytoplasm to the plasma membrane of endothelial cells, where it is exposed to the immune system and becomes the target of autoimmune responses [10]. To confirm and extend our previous data, in the present study, we evaluated whether oxidative stress induced by H_2_O_2_ treatment upregulates surface expression of HSP90 in primary human endothelial cells, a more physiological cellular *in vitro* system, and whether this event implies an alteration of HSP90 gene expression, cellular distribution, and secretion.

Given the role of oxidative stress in the pathogenesis and progression of atherosclerotic disease, including the activation of autoimmune responses, we also wanted to determine the ability of the polyphenolic antioxidant compound 7,8-dihydroxy-4-methylcoumarin (7,8-DHMC) to prevent the effects of oxidative stress on HSP90 cellular distribution and secretion.

## 2. Material and Methods

### 2.1. Cell Cultures

Human umbilical vein endothelial cells (HUVEC; Clonetics/Lonza, Basel, Switzerland) were grown in endothelial cell growth medium (EGM-2; Lonza), at 37°C in a humidified atmosphere with 5% CO_2_/95% air, as previously described [12] and were used between passages 3 and 5. To expose HUVEC to a prooxidant microenvironment, 5 × 10^4^ cells/ml was cultured in endothelial cell basal medium (EBM-2; Lonza) containing 100, 300, 600, and 800 *μ*M H_2_O_2_ (Sigma, St. Louis, MO, USA) or medium alone. After 1 hour, the culture medium was substituted with EGM-2, and cells were incubated for 2 and 4 hours (recovery phase) (1, 4, 8, and 24 hours for mRNA quantification experiments).

To determine the effects of 7,8-DHMC on H_2_O_2_-mediated alteration of HSP90 distribution, 7,8-DHMC was prepared as previously described [13] and dissolved in dimethyl sulfoxide (DMSO; Sigma) at the concentration of 60 mg/ml. This preparation was diluted in EBM-2 to obtain the final concentrations of 1 *μ*g/ml (1 : 60,000) and 10 *μ*g/ml (1 : 6000) used to pretreat HUVEC. In our preliminary experiments, these concentrations resulted the most efficacious to prevent HSP90 surface expression. Of note, these concentrations are below the maximal tolerable dose (100 *μ*g/ml) not affecting cell viability, as demonstrated in a previous study by trypan blue exclusion test and by microscopic examination of cell morphology [13]. HUVEC pretreated with 7,8-DHMC diluent alone (DMSO diluted 1 : 6000 in EBM-2) were used as controls. After 30-minute pretreatment with 7,8-DHMC, HUVEC were exposed to 600 *μ*M H_2_O_2_ as previously described.

### 2.2. Flow Cytometry

To evaluate the surface expression of HSP90, HUVEC were labelled with a rabbit polyclonal antihuman HSP90 antibody (Ab; Santa Cruz Biotechnology Inc., Texas, USA). After being washed, cells were incubated with Alexa Fluor 488-conjugated goat antirabbit IgG Ab (Invitrogen Molecular Probes, Carlsbad, CA, USA) and fixed with 1% paraformaldehyde.

To evaluate intracellular expression of HSP90, HUVEC were incubated with fluorescence-activated cell sorted (FACS) permeabilizing solution (Becton Dickinson Co., CA, USA) and then labelled as described for surface expression. Samples labelled with only antirabbit IgG-Alexa Fluor 488 (Invitrogen Molecular Probes) were used as negative controls. Samples were analyzed using a FACSCalibur flow cytometer (Becton Dickinson) and CellQuest software (Becton Dickinson).

### 2.3. Immunofluorescence Analysis and Confocal Microscopy

To evaluate H_2_O_2_-induced modifications of HSP90 distribution within endothelial cells, HUVEC were fixed with 4% formaldehyde for 30 minutes and permeabilized with Triton X-100 0.1% (v/v). After being washed, cells were incubated with rabbit polyclonal antihuman HSP90 Ab (Santa Cruz Biotechnology Inc.) followed by Alexa Fluor 488-conjugated goat antirabbit IgG Ab (Invitrogen Molecular Probes). Samples labelled with only antirabbit IgG-Alexa Fluor 488 Ab were used as negative controls. All samples were counterstained with Hoechst 33342 (Molecular Probes), mounted with glycerol-PBS (2 : 1), and analyzed by using a high-resolution objective (63x) through a confocal laser scanning microscope Zeiss LSM 510 equipped with argon and HeNe ion lasers.

### 2.4. Semiquantitative Reverse-Transcription Polymerase Chain Reaction (qRT-PCR)

To evaluate HSP90 gene expression, total cellular RNA was extracted from HUVEC using the TRIzol Reagent (Invitrogen-Life Technologies Italia, Monza, Italy) as previously described [14]. First-strand cDNAs were synthesized using a mixture of 2 *μ*g of total RNA, oligo(dT)_12–18_ primers, and SuperScript III Reverse Transcriptase (Invitrogen). qRT-PCR analysis was carried out using Power SYBR Green PCR Master Mix (QIAGEN, Hilden, Germany) and quantified with the ABI PRISM 7000 SDS (Applied Biosystems, Monza, Italy). Relative expression was calculated using the comparative cycle threshold (Ct) method (2^−ΔΔCt^). mRNA expression was normalized for beta-2 microglobulin levels. The following primers were used for human HSP90: forward CTTGGGTCTGGGTTTCCTC; reverse GGGCAACACCTCTACAAGGA.

### 2.5. ELISA

Soluble HSP90 (sHSP90) concentrations in culture supernatants were quantified by a commercially available ELISA kit (Cusabio Biotech Co. Ltd., Wuhan, Hubei, China). The minimum detectable dose of the assay was less than 3.9 pg/ml.

### 2.6. Statistical Analysis

Results are expressed as arithmetic mean values and standard error of the mean. Data were tested for Gaussian distribution with the Kolmogorov–Smirnov test and analyzed using one-way ANOVA with a Bonferroni post hoc test or Student's *t*-test.

Correlations were tested with Spearman and Pearson correlation coefficient upon normality test carried out by the D'Agostino and Pearson omnibus normality test, and the two-phase decay was used to obtain the fit. Statistical analyses were performed using GraphPad Prism 5 software (GraphPad Software Inc., La Jolla, CA, USA). A *P* value less than 0.05 was considered statistically significant.

## 3. Results

### 3.1. H_2_O_2_ Treatment Upregulates HSP90 Surface Expression

**Figure 1:**
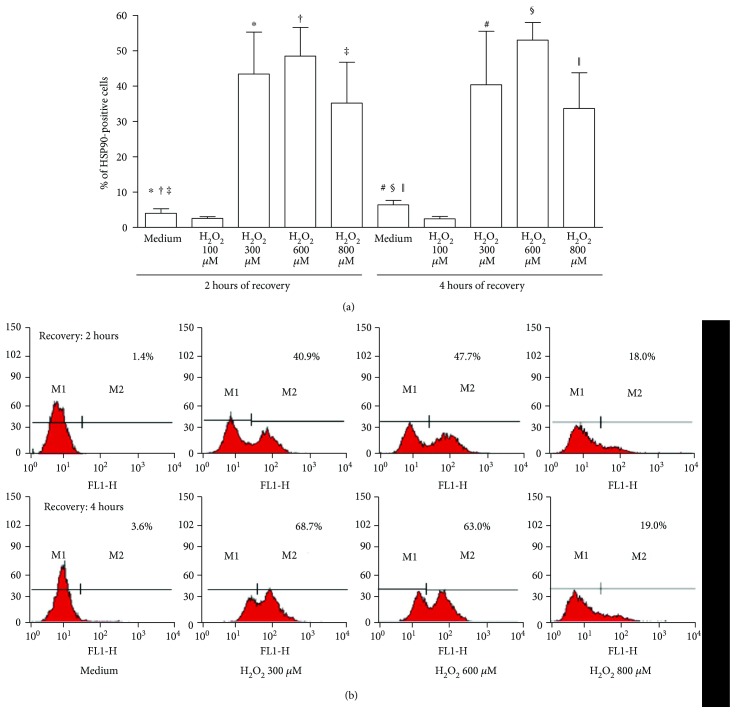
Cytofluorimetric analysis of HSP90 surface expression (percentages of positive cells) in HUVEC treated with H_2_O_2_ or left untreated. (a) Results expressed as mean percentages and standard error of the mean (*n* = 6). ^∗^*P* = 0.0121; ^†^*P* = 0.0061; ^‡^*P* = 0.0061; ^#^*P* = 0.0249; ^§^*P* < 0.0001; ^║^*P* = 0.0064. (b) Representative histograms showing HSP90 surface expression in HUVEC treated with 300, 600, and 800 *μ*M of H_2_O_2_ or left untreated. M1: negative cells; M2: positive cells. Samples labelled with only antirabbit IgG-Alexa Fluor 488 antibody were used as negative controls of the cytofluorimetric assay.

Cytofluorimetric analysis showed a significant increase in the percentage of HUVEC positive for surface expression of HSP90 when cells were treated with 300, 600, and 800 *μ*M H_2_O_2_ (2 hours of recovery: 300 *μ*M *P* = 0.0121, 600 *μ*M *P* = 0.0061, 800 *μ*M *P* = 0.0061; 4 hours of recovery: 300 *μ*M *P* = 0.0249, 600 *μ*M *P* < 0.0001, 800 *μ*M *P* = 0.0064) (Figures 1(a) and 1(b)). Analysis of HSP90 intracellular expression did not show any significant change in cells treated with H_2_O_2_ except for a slight reduction of the mean fluorescence intensity (MFI) after 2 hours of cell recovery (data not shown).

### 3.2. H_2_O_2_ Treatment Reduces HSP90 Secretion

**Figure 2:**
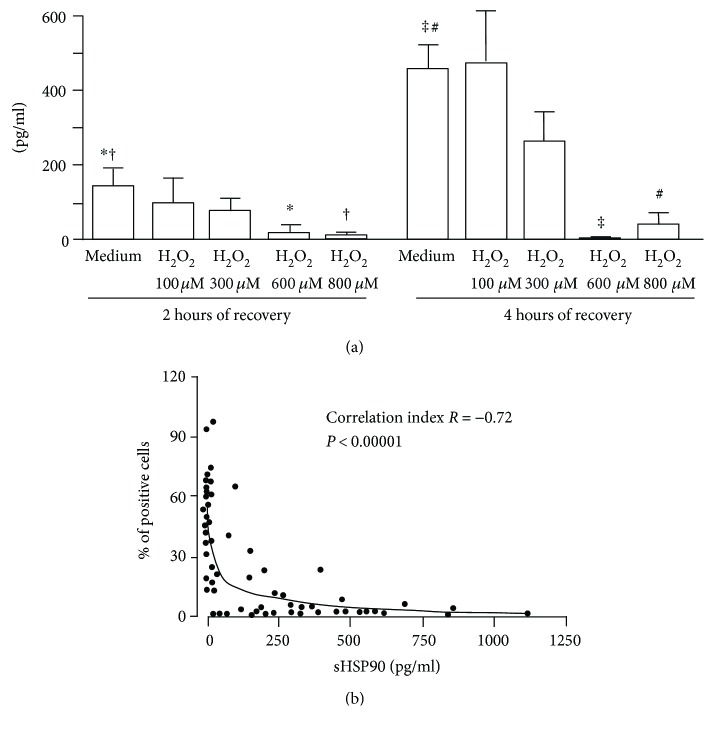
(a) Analysis of soluble HSP90 concentrations (pg/ml) by ELISA in culture supernatants from HUVEC treated with H_2_O_2_ or left untreated. Results are expressed as mean and standard error of the mean (*n* = 6). ^∗^*P* = 0.0109; ^†^*P* = 0.0162; ^‡^*P* = 0.0010; ^#^*P* = 0.0008. (b) Spearman correlation between the percentages of HSP90-positive cells evaluated by cytofluorimetric assay, and the concentrations of soluble HSP90 (pg/ml) in culture supernatants determined by ELISA in HUVEC treated with H_2_O_2_ or left untreated after 2 and 4 hours of recovery. Spearman correlation was chosen since a non-Gaussian distribution of the data was observed according to the D'Agostino and Pearson omnibus normality test. The two-phase decay was used to obtain the fit.

Analysis of sHSP90 concentration in culture supernatants by ELISA demonstrated a reduced secretion of this protein by HUVEC when treated with H_2_O_2_ compared to untreated cells (2 hours of recovery: 600 *μ*M *P* = 0.0109, 800 *μ*M *P* = 0.0162; 4 hours of recovery: 600 *μ*M *P* = 0.0010, 800 *μ*M *P* = 0.0008) (Figure 2(a)). Of note, a significant negative correlation was observed between the percentage of surface HSP90-positive cells and the concentrations of sHSP90 obtained from all samples at 2 and 4 hours of recovery (*r* = −0.72, *P* < 0.00001) (Figure 2(b)).

### 3.3. 7,8-DHMC Prevents Surface HSP90 Upregulation Induced by Oxidative Stress

**Figure 3:**
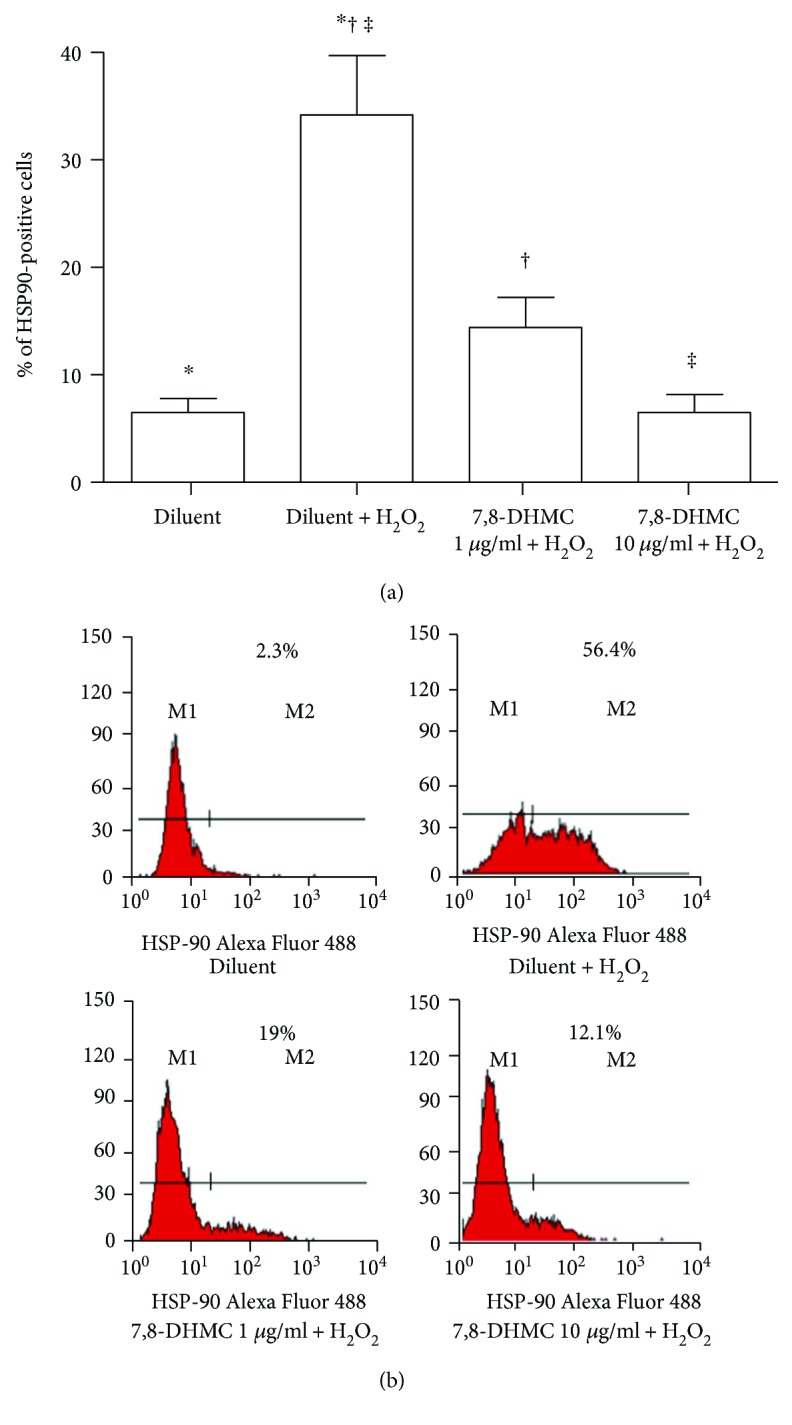
Cytofluorimetric analysis of HSP90 surface expression in HUVEC pretreated with 7,8-dihydroxy-4-methylcoumarin (7,8-DHMC) or diluent alone as control and then exposed to H_2_O_2_. (a) Results are expressed as mean percentages and standard error of the mean (*n* = 6). ^∗^*P* < 0.001; ^†^*P* < 0.01; ^‡^*P* < 0.001. (b) Representative histograms. M1: negative cells; M2: positive cells. Samples labelled with only antirabbit IgG-Alexa Fluor 488 antibody were used as negative controls of the cytofluorimetric assay.

When HUVEC were pretreated with 1 and 10 *μ*g/ml of 7,8-DHMC before exposure to 600 *μ*M of H_2_O_2_, the percentage of cells positive for HSP90 surface expression was significantly lower than that observed of cells exposed to H_2_O_2_ and not pretreated with 7,8-DHMC (1 *μ*g/ml *P* < 0.01; 10 *μ*g/ml *P* < 0.001) (Figures 3(a) and 3(b)). The corresponding percentages of inhibition were 40% (1 *μ*g/ml 7,8-DHMC) and 80% (10 *μ*g/ml 7,8-DHMC).

**Figure 4:**
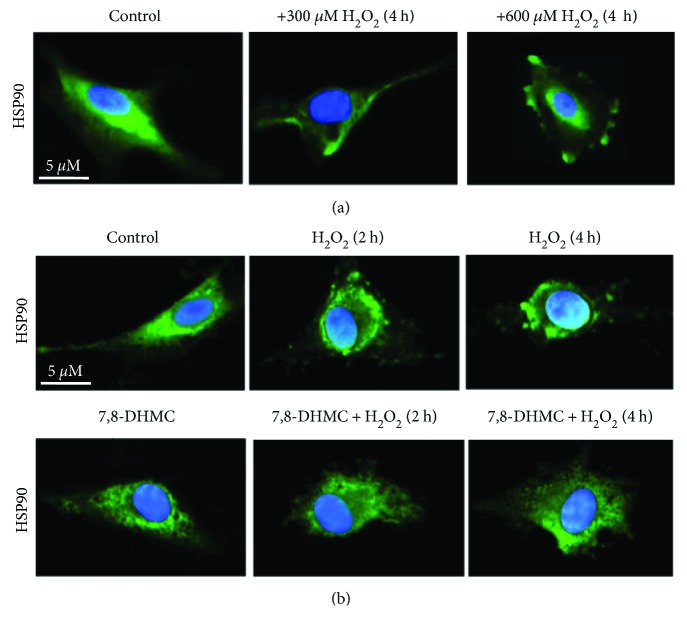
(a) Dose-dependent effect of H_2_O_2_ on cellular HSP90 distribution in HUVEC. (b) Cellular HSP90 distribution in HUVEC pretreated with 7,8-dihydroxy-4-methylcoumarin (7,8-DHMC) or diluent alone as control and then exposed to 600 *μ*M of H_2_O_2_ or left untreated. Images obtained by scanning confocal microscopy analysis were collected at 512 × 512 pixels. Green: HSP90 staining; blue: nucleus staining.

The effect of 7,8-DHMC pretreatment on HSP90 cellular expression was also evaluated by both indirect immunofluorescence with scanning confocal microscopy analysis and qRT-PCR. Immunofluorescence showed a prevalent cytoplasmic distribution of the protein in untreated cells and a preferential increased surface localization in HUVEC treated with 300 and 600 *μ*M H_2_O_2_ (Figure 4(a)), thus confirming cytofluorimetric results (Figure 1). Again, pretreatment of HUVEC with 10 *μ*g/ml 7,8-DHMC prevented surface HSP90 localization in H_2_O_2_-treated cells (600 *μ*M) at 2 and 4 hours, respectively, as clearly shown in Figure 4(b), bottom panels.

qRT-PCR demonstrated that HSP90 gene expression in cells exposed to H_2_O_2_, pretreated or not with 7,8-DHMC, was not significantly different from that observed in untreated cells (data not shown).

**Figure 5:**
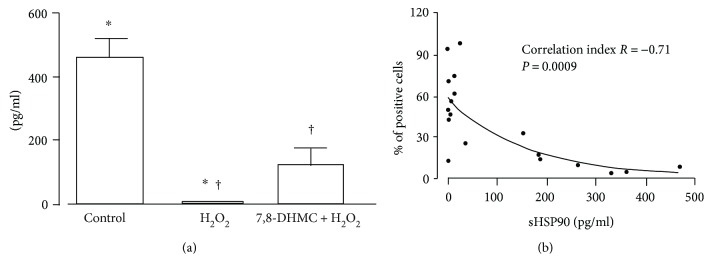
(a) Analysis of soluble HSP90 concentrations (pg/ml) by ELISA in culture supernatants from HUVEC pretreated with 7,8-dihydroxy-4-methylcoumarin (7,8-DHMC) or diluent alone and then exposed to H_2_O_2_ or left untreated. Results are expressed as mean and standard error of the mean (*n* = 3). ^∗^*P* < 0.001; ^†^*P* = 0.037. (b) Pearson correlation between the percentages of surface HSP90-positive cells evaluated by cytofluorimetric assay and the concentrations of soluble HSP90 (pg/ml) in culture supernatants determined by ELISA in HUVEC pretreated with 7,8-dihydroxy-4-methylcoumarin (7,8-DHMC) or diluent alone and then exposed to H_2_O_2_ or left untreated. Pearson correlation was chosen since a Gaussian distribution of the data was observed according to the D'Agostino and Pearson omnibus normality test. The two-phase decay was used to obtain the fit.

When we examined HSP90 secretion in culture medium, we observed that pretreatment of cells with 7,8-DHMC partially prevented the inhibition of HSP90 secretion in culture supernatants caused by H_2_O_2_ exposure (*P* = 0.037) (Figure 5(a)). A significant negative correlation was observed between the surface expression of HSP90 and its secretion (*r* = −0.71, *P* = 0.0009) (Figure 5(b)).

## 4. Discussion

The results of this study demonstrate that a prooxidant microenvironment upregulates HSP90 expression on primary human endothelial cell membrane. This event does not imply an increased HSP90 gene expression or a decreased intracytoplasmic protein concentration, whereas it is associated with a decreased protein secretion. The antioxidant 7,8-DHMC is able to counteract the effects of oxidative stress on HSP90 cellular distribution and secretion, thus preventing a possible exposure of the protein to the immune system at the endothelial cell surface.

We had previously observed that oxidative stress was able to induce surface expression of HSP90 in the immortalized endothelial cell line EAhy-926 [10]. Our results here extend these previous observations to primary endothelial cells that represent a more physiological *in vitro* system and are in accordance with previous findings on oxidative stress-induced HSP90 surface expression in dog neutrophils treated with H_2_O_2_ [15]. Our results are also in line with previous observations of HSP90 surface expression in lymphocytes and monocytes of patients with systemic lupus erythematosus [16] and in aortic endothelium of diabetic rats [17].

The process regulating surface expression and release of HSPs, which lacks the consensus peptide for membrane translocation, is complex and not completely understood, and different alternative pathways have been proposed [6].

In our study, we observed that oxidative stress triggers in endothelial cells molecular pathways that result in increased HSP90 surface expression and reduced protein secretion. The finding on reduced HSP90 secretion in response to H_2_O_2_ treatment is in contrast with previous observation by Liao et al. on increased HSP90 release by rat vascular smooth muscle cells under oxidative stress [18]. The discrepancies between our results and those of Liao et al. could be ascribed to different cell types and experimental conditions. In particular, Liao and colleagues used rat-derived vascular smooth muscle cells and not human endothelial cells. Furthermore, they used the naphthoquinolinedione LY83583, a generator of O⨪2, as prooxidant compound instead of H_2_O_2_.

It is well known that HSPs have cytoprotective roles when located in the cytosol, whereas they provide danger signals for the immune system when secreted or exposed on the plasma membrane [19]. Surface exposure of HSP90 induced by antitumor drugs on dying myeloma cells promotes their uptake by dendritic cells and the induction of specific immune responses to tumor cells [20]. Similarly, the proinflammatory and prooxidant conditions of atherosclerotic plaques, by inducing protein cell surface localization, can promote HSP90 immunogenicity. Other HSPs, such as HSP60, have been already implicated as candidate autoantigens in the development and progression of atherosclerosis. T lymphocytes able to recognize HSP60 epitopes have been identified in early and advanced plaques [21]. Furthermore, it has been demonstrated that antibody levels against HSP60/65 are increased in subjects with established cardiovascular disease and predict further development of the pathology [22]. We have previously demonstrated that overexpression of HSP90 in human atherosclerotic plaques is accompanied by the presence of HSP90-specific T lymphocytes with a predominant Th1 proinflammatory profile, possibly involved in the thrombogenicity of the atherosclerotic lesion [10, 11]. We have also detected the presence of anti-HSP90-specific serum autoantibodies in about half of the patients with carotid atherosclerosis [10, 11]. It is known that autoantibodies may cause tissue damage by immune-complex deposition, by complement activation, or by binding soluble or membrane proteins, thus blocking or activating their biological activities [23, 24]. Previous papers demonstrated that stressed endothelial cells are lysed by HSP60-specific antibodies [25] and that anti-HSP60 autoantibodies in sera from patients with systemic lupus erythematosus induce HUVEC apoptosis [26]. Of interest, a very recent paper by Zhang and colleagues demonstrated that the presence of HSP90 on the cell membrane protects membrane phospholipids from oxidation induced by H_2_O_2_ [27]. This finding obtained *in vitro* by using liposomes and *E. coli* strongly suggests that HSP90 surface expression in HUVEC exposed to oxidative stress could be a defence mechanism of endothelial cells to protect membrane integrity, thus counteracting lipid peroxidation and cell apoptosis caused by oxidative stress [28, 29]. On the basis of this recent evidence, we can hypothesize that autoantibodies to HSP90 can bind the specific HSP on the endothelial cell surface (as reported for HSP60-specific antibodies) and affect the ability of this protein to protect endothelial cells from oxidative stress damage and apoptosis.

Due to the leading role played by oxidative stress in the promotion of endothelial dysfunction in atherosclerosis, treatment with antioxidant agents may have protective effects in this disease [30, 31]. We focused our interest on coumarins, natural compounds identified in plants, fungi, and bacteria, which exhibit antimicrobial, anti-inflammatory, and antioxidant properties [32–35]. Previous results demonstrated that coumarins have a protective role against cytotoxicity induced by H_2_O_2_ in HUVEC by reducing lipid peroxidation and ROS levels and upregulating antiapoptotic genes [34].

In the present study, we evaluated the effects of 7,8-DHMC on oxidative stress-induced modifications of HSP90 cellular distribution. We have chosen this antioxidant because the 4-methylcoumarins do not generate toxic coumarin 3,4-epoxides during their metabolism in the liver [36] and because the presence of two hydroxyl groups increases the antioxidant and anti-inflammatory activities of coumarins [13]. Previous studies demonstrated that the antioxidant activity of 7,8-DHMC is related, at least in part, to efficient scavenging of the oxygen radicals [37] and to inhibition of oxygen radical formation [38].

Our results here show that pretreatment with 7,8-DHMC is able to efficiently prevent the effects of oxidative stress on HSP90 surface expression and secretion in human endothelial cells and suggest that this compound could be useful to prevent the possible exposure of HSP90 to the immune system on endothelial cell surface. It remains to demonstrate a possible pathogenic effect mediated by surface HSP90 interaction with the immune system and to assess the intracellular signalling pathway underlying the effects of H_2_O_2_ on HSP90, which will be the object of a future work.

## 5. Conclusions

Overall, our study demonstrates for the first time that primary human endothelial cells exposed to a strong oxidative stress express surface HSP90 that becomes accessible to the immune system and a possible target of autoreactive responses. We can speculate that in the prooxidant conditions of atherosclerotic plaque, the expression of HSP90 on endothelial cell surface and the presence of vascular-associated dendritic cells in the aortic adventitia [11, 39] may create a microenvironment predisposed to the induction of a local autoimmune response which contributes to endothelial damage. 7,8-DHMC, due to its ability to hamper oxidative stress-induced HSP90 surface expression, could be useful to prevent possible autoimmune mechanisms involving HSP90. The low toxicity and the wide distribution in food plants of coumarins [40] make these compounds promising tools to contrast prooxidant, proinflammatory, and autoimmune mechanisms in humans.

## References

[1] Nilsson J., Hansson G. K. (2008). Autoimmunity in atherosclerosis: a protective response losing control?. *Journal of Internal Medicine*.

[2] Kimura T., Tse K., Sette A., Ley K. (2015). Vaccination to modulate atherosclerosis. *Autoimmunity*.

[3] Sriram K., Rodriguez-Fernandez M., Doyle F. J. (2012). A detailed modular analysis of heat-shock protein dynamics under acute and chronic stress and its implication in anxiety disorders. *PLoS One*.

[4] Mehta T. A., Greenman J., Ettelaie C., Venkatasubramaniam A., Chetter I. C., McCollum P. T. (2005). Heat shock proteins in vascular disease—a review. *European Journal of Vascular and Endovascular Surgery*.

[5] Lee S., Park Y., Zuidema M. Y., Hannink M., Zhang C. (2011). Effects of interventions on oxidative stress and inflammation of cardiovascular diseases. *World Journal of Cardiology*.

[6] De Maio A. (2011). Extracellular heat shock proteins, cellular export vesicles, and the stress observation system: a form of communication during injury, infection, and cell damage. It is never known how far a controversial finding will go! Dedicated to Ferruccio Ritossa. *Cell Stress and Chaperones*.

[7] Tukaj S., Węgrzyn G. (2016). Anti-Hsp90 therapy in autoimmune and inflammatory diseases: a review of preclinical studies. *Cell Stress and Chaperones*.

[8] Haque A., Alam Q., Alam M. Z. (2016). Current understanding of HSP90 as a novel therapeutic target: an emerging approach for the treatment of cancer. *Current Pharmaceutical Design*.

[9] Tukaj S., Zillikens D., Kasperkiewicz M. (2015). Heat shock protein 90: a pathophysiological factor and novel treatment target in autoimmune bullous skin diseases. *Experimental Dermatology*.

[10] Riganò R., Profumo E., Buttari B. (2007). Heat shock proteins and autoimmunity in patients with carotid atherosclerosis. *Annals of the New York Academy of Sciences*.

[11] Businaro R., Profumo E., Tagliani A. (2009). Heat-shock protein 90: a novel autoantigen in human carotid atherosclerosis. *Atherosclerosis*.

[12] D'Arcangelo D., Gaetano C., Capogrossi M. C. (2002). Acidification prevents endothelial cell apoptosis by Axl activation. *Circulation Research*.

[13] Kumar S., Singh B. K., Kalra N. (2005). Novel thiocoumarins as inhibitors of TNF-*α* induced ICAM-1 expression on human umbilical vein endothelial cells (HUVECs) and microsomal lipid peroxidation. *Bioorganic & Medicinal Chemistry*.

[14] D'Arcangelo D., Facchiano F., Nassa G. (2016). PDGFR-alpha inhibits melanoma growth via CXCL10/IP-10: a multi-*omics* approach. *Oncotarget*.

[15] Camins A., Diez-Fernandez C., Prieto P. (1999). Cell-surface expression of heat shock proteins in dog neutrophils after oxidative stress. *Toxicology In Vitro*.

[16] Erkeller-Yüksel F. M., Isenberg D. A., Dhillon V. B., Latchman D. S., Lydyard P. M. (1992). Surface expression of heat shock protein 90 by blood mononuclear cells from patients with systemic lupus erythematosus. *Journal of Autoimmunity*.

[17] Lei H., Romeo G., Kazlauskas A. (2004). Heat shock protein 90*α*-dependent translocation of annexin II to the surface of endothelial cells modulates plasmin activity in the diabetic rat aorta. *Circulation Research*.

[18] Liao D. F., Jin Z. G., Baas A. S. (2000). Purification and identification of secreted oxidative stress-induced factors from vascular smooth muscle cells. *The Journal of Biological Chemistry*.

[19] Pockley A. G. (2003). Heat shock proteins as regulators of the immune response. *The Lancet*.

[20] Spisek R., Charalambous A., Mazumder A., Vesole D. H., Jagannath S., Dhodapkar M. V. (2007). Bortezomib enhances dendritic cell (DC)-mediated induction of immunity to human myeloma via exposure of cell surface heat shock protein 90 on dying tumor cells: therapeutic implications. *Blood*.

[21] Almanzar G., Öllinger R., Leuenberger J. (2012). Autoreactive HSP60 epitope-specific T-cells in early human atherosclerotic lesions. *Journal of Autoimmunity*.

[22] Xu Q., Kiechl S., Mayr M. (1999). Association of serum antibodies to heat-shock protein 65 with carotid atherosclerosis: clinical significance determined in a follow-up study. *Circulation*.

[23] Margutti P., Matarrese P., Conti F. (2008). Autoantibodies to the C-terminal subunit of RLIP76 induce oxidative stress and endothelial cell apoptosis in immune-mediated vascular diseases and atherosclerosis. *Blood*.

[24] Martin F., Chan A. C. (2004). Pathogenic roles of B cells in human autoimmunity; insights from the clinic. *Immunity*.

[25] Grundtman C., Kreutmayer S. B., Almanzar G., Wick M. C., Wick G. (2011). Heat shock protein 60 and immune inflammatory responses in atherosclerosis. *Arteriosclerosis, Thrombosis, and Vascular Biology*.

[26] Dieudé M., Senécal J. L., Raymond Y. (2004). Induction of endothelial cell apoptosis by heat-shock protein 60-reactive antibodies from anti-endothelial cell autoantibody-positive systemic lupus erythematosus patients. *Arthritis and Rheumatism*.

[27] Zhang M., Wang D., Geng Z., Li P., Sun Z., Xu W. (2018). Effect of heat shock protein 90 against ROS-induced phospholipid oxidation. *Food Chemistry*.

[28] Choudhary S., Zhang W., Zhou F. (2002). Cellular lipid peroxidation end-products induce apoptosis in human lens epithelial cells. *Free Radical Biology & Medicine*.

[29] Ayala A., Muñoz M. F., Argüelles S. (2014). Lipid peroxidation: production, metabolism, and signaling mechanisms of malondialdehyde and 4-hydroxy-2-nonenal. *Oxidative Medicine and Cellular Longevity*.

[30] Yamashita S., Matsuzawa Y. (2009). Where are we with probucol: a new life for an old drug?. *Atherosclerosis*.

[31] Rosenblat M., Hayek T., Aviram M. (2006). Anti-oxidative effects of pomegranate juice (PJ) consumption by diabetic patients on serum and on macrophages. *Atherosclerosis*.

[32] Morabito G., Trombetta D., Singh Brajendra K. (2010). Antioxidant properties of 4-methylcoumarins in *in vitro* cell-free systems. *Biochimie*.

[33] Drábiková K., Perečko T., Nosál' R., Harmatha J., Smidrkal J., Jančinová V. (2013). Study of possible mechanisms involved in the inhibitory effects of coumarin derivatives on neutrophil activity. *Oxidative Medicine and Cellular Longevity*.

[34] Whang W. K., Park H. S., Ham I. (2005). Natural compounds, fraxin and chemicals structurally related to fraxin protect cells from oxidative stress. *Experimental & Molecular Medicine*.

[35] Joshi R., Kumar A., Manral S. (2013). Calreticulin transacetylase mediated upregulation of thioredoxin by 7,8-diacetoxy-4-methylcoumarin enhances the antioxidant potential and the expression of vascular endothelial growth factor in peripheral blood mononuclear cells. *Chemico-Biological Interactions*.

[36] Vassallo J. D., Hicks S. M., Born S. L., Daston G. P. (2004). Roles for epoxidation and detoxification of coumarin in determining species differences in Clara cell toxicity. *Toxicological Sciences*.

[37] Raj H. G., Parmar V. S., Jain S. C. (1998). Mechanism of biochemical action of substituted 4-methylbenzopyran-2-ones. Part I: dioxygenated 4-methyl coumarins as superb antioxidant and radical scavenging agents. *Bioorganic & Medicinal Chemistry*.

[38] Raj H. G., Sharma R. K., Garg B. S. (1998). Mechanism of biochemical action of substituted 4-methylbenzopyran-2-ones. Part 3: a novel mechanism for the inhibition of biological membrane lipid peroxidation by dioxygenated 4-methylcoumarins mediated by the formation of a stable ADP-Fe-inhibitor mixed ligand complex. *Bioorganic & Medicinal Chemistry*.

[39] Millonig G., Schwentner C., Mueller P., Mayerl C., Wick G. (2001). The vascular-associated lymphoid tissue: a new site of local immunity. *Current Opinion in Lipidology*.

[40] Lake B. G. (1999). Coumarin metabolism, toxicity and carcinogenicity: relevance for human risk assessment. *Food and Chemical Toxicology*.

